# Anti-infective activity of apolipoprotein domain derived peptides *in vitro*: identification of novel antimicrobial peptides related to apolipoprotein B with anti-HIV activity

**DOI:** 10.1186/1471-2172-11-13

**Published:** 2010-03-18

**Authors:** Bridie A Kelly, Ian Harrison, Áine McKnight, Curtis B Dobson

**Affiliations:** 1 Faculty of Life Sciences, Stopford Building, The University of Manchester, Manchester, M13 9PT, UK; 2 Queen Mary University of London, School of Medicine and Dentistry, Centre for Immunology & Infectious Disease, Blizard Institute of Cell and Molecular Science, Barts & The London, 4 Newark Street, Whitechapel, London, E1 2AT, UK

## Abstract

**Background:**

Previous reports have shown that peptides derived from the apolipoprotein E receptor binding region and the amphipathic α-helical domains of apolipoprotein AI have broad anti-infective activity and antiviral activity respectively. Lipoproteins and viruses share a similar cell biological niche, being of overlapping size and displaying similar interactions with mammalian cells and receptors, which may have led to other antiviral sequences arising within apolipoproteins, in addition to those previously reported. We therefore designed a series of peptides based around either apolipoprotein receptor binding regions, or amphipathic α-helical domains, and tested these for antiviral and antibacterial activity.

**Results:**

Of the nineteen new peptides tested, seven showed some anti-infective activity, with two of these being derived from two apolipoproteins not previously used to derive anti-infective sequences. Apolipoprotein J (151-170) - based on a predicted amphipathic alpha-helical domain from apolipoprotein J - had measurable anti-HSV1 activity, as did apolipoprotein B (3359-3367) dp (apoBdp), the latter being derived from the LDL receptor binding domain B of apolipoprotein B. The more active peptide - apoBdp - showed similarity to the previously reported apoE derived anti-infective peptide, and further modification of the apoBdp sequence to align the charge distribution more closely to that of apoEdp or to introduce aromatic residues resulted in increased breadth and potency of activity. The most active peptide of this type showed similar potent anti-HIV activity, comparable to that we previously reported for the apoE derived peptide apoEdpL-W.

**Conclusions:**

These data suggest that further antimicrobial peptides may be obtained using human apolipoprotein sequences, selecting regions with either amphipathic α-helical structure, or those linked to receptor-binding regions. The finding that an amphipathic α-helical region of apolipoprotein J has antiviral activity comparable with that for the previously reported apolipoprotein AI derived peptide 18A, suggests that full-length apolipoprotein J may also have such activity, as has been reported for full-length apolipoprotein AI. Although the strength of the anti-infective activity of the sequences identified was limited, this could be increased substantially by developing related mutant peptides. Indeed the apolipoprotein B-derived peptide mutants uncovered by the present study may have utility as HIV therapeutics or microbicides.

## Background

A number of studies have reported molecular interactions between viruses and lipoproteins (LPs), apolipoproteins or their receptors, with an underlying basis for these links being the similarities in location, size and function of LPs and virus particles (reviewed in [1]). One of the most studied such connections to date is the influence of the APOE gene on the outcome of infection [2-7]. APOE codes for the protein apolipoprotein E (apoE), which like other apolipoproteins is a constituent of LP particles. We have shown that the influence of APOE on infection may be mediated through direct anti-infective activity of the cationic receptor-binding region of apolipoprotein E (apoE141-149). We reported that tandem repeat peptides derived from this region - in particular apoE(141-149)dp (apoEdp) - and N-terminal truncated apoE-4 have broad antiviral activity *in vitro *[8,9]. Recently another group have shown such peptides have direct antiviral activity *in vivo*, reducing HSV1 titres in an experimental herpes simplex keratitis model [10].

We have suggested that the broad antiviral action of this heparan sulphate proteoglycan (HSPG) and low density lipoprotein receptor (LDLR) binding region may relate to the ubiquity of the former receptor as an initial attachment site to cells for many viruses, along with the ability of some viruses to enter cells using LDLR family receptors. Thus apoE may compete for binding to these receptors preventing viral attachment [1]. In addition apoEdp and related peptides exert a virucidal activity on viruses and also show broad antibacterial activity, which may more closely relate to the high numbers of cationic and basic hydrophobic residues within apoEdp and related peptides [8,9]. It is at present unclear whether full-length or N-terminal truncated apoE proteins also show such antibacterial activity.

Other researchers have focused on antiviral activity of peptides derived from another apolipoprotein - apolipoprotein AI (apoAI) - with a peptide referred to as 18A being shown to block fusion of virus particles to cells. This peptide is a consensus domain constructed from the 22 mer amphipathic helical domains within apoAI - and its activity appears to involve its amphipathic -helical structure leading to blockade of specific viral envelope proteins [11].

The biophysical properties of apoE-derived or apoAI-derived anti-infective peptides are shared by similar regions within other apolipoproteins. In particular many other apolipoproteins contain amphipathic -helical regions. Apolipoprotein AII (apoAII) contains a region - apoAII(18-30) - which has previously been stabilised as an α-helical peptide by addition of a 5 mer motif to the C-terminal to promote α-helical structure [12]. In a separate study apolipoprotein J (apoJ) was predicted to contain five amphipathic -helical regions, which together allow this apolipoprotein to act as a biological detergent [13].

Similarly several other apolipoprotein regions contain binding domains analogous to the region we previously investigated in apoE. A peptide based on a domain within apolipoprotein B (apoB) - apoB(1000-1016) - has been reported to function as an arterial binding domain [14]. ApoB also contains two LDL receptor binding domains comparable to the receptor binding region of apoE, namely region A (apoB3147-3157) - and region B (apoB3359-3367). As region B is more uniformly conserved across species it has been considered to be more important for receptor binding [15]. Previous studies have shown that heparin-binding activity within apolipoprotein H (apoH or beta-2-glycoprotein) depends on three Lys residues in its fifth domain (in positions 284, 286, and 287 of apoH), and that an octomer peptide apoH_281-288 _competitively inhibits apoH binding to heparin [16]. Finally a second heparin-binding region with apoE has been linked to residues 211-218 [17].

Given the ready access of LPs to extracellular pathogens, and the existence of many domains in apolipoproteins similar to the two previously reported to have anti-infective activity, in the present study we tested the potential activity of a variety of apolipoprotein-derived peptides, grouped by similarity to the previously reported apoE or apoAI anti-infective peptides. Specifically we looked at either (i) possible antiviral action of amphipathic α-helical peptides (comparable with the apoA1-derived 18A peptide) or (ii) broader anti-infective activity of tandem repeats of receptor binding domains (similar to the cationic non-amphipathic α-helical apoE peptides).

The first group of peptides included several of the 22 mer amphipathic alpha helical domains from apoA1 (which had never previously been tested) [11], apoAII 18-30 with the artificial helix promoting region (apoAII(18-30)+) or the equivalent peptide containing an additional five residues from the apoAII sequence (apoAII(18-35)). We also synthesised peptide mimetics of four of the predicted amphipathic helical domains of apoJ [13]. In the second group we examined the activities of apoB(1000-1016), apoB(3359-3367)dp, apoH(281-288)dp and apoE(211-219)dp and apoE(213-221)dp, along with some tandem repeats and non-repeat peptides related to the apoE(141-149) region. In addition we examined the activity of apoE(141-149)dp derived from either the bovine or murine apolipoprotein E sequence. Tandem repeat peptides were synthesised to allow the structure of these short regions to more likely mimic that found in the full length protein, using the strategy we previously utilised for apoE(141-149) derived peptides [18] and for other heparin-binding domain related AMPs [19]. Finally we devised and tested mutant peptides related to apoB(3359-3368)dp, modified to more closely resemble the apoE-derived peptide mutants we previously reported [9].

## Results

### Anti-infective activity of apolipoprotein-derived amphipathic α-helical peptides

All peptides were tested for activity against HSV1, the Gram positive bacterium *Staphylococcus aureus *and the Gram negative bacterium *Pseudomonas aeruginosa*, and resulting data are summarised in Table 1. The apoE-derived peptides, showed most consistent activity, although some anti-infective activity was apparent in other apolipoprotein derived sequences. As expected, the positive control apoA1-derived peptide 18A showed antiviral activity, with an IC50 concentration against HSV1 of 36.5 μM (95%CI 31.9 - 41.1 μM). No antibacterial activity was apparent suggesting a relatively selective mechanism of action. None of the apoAI amphipathic helical domains from which the 18A peptide was derived, showed any antiviral activity (these had not been directly tested in previous reports [11,20,21]). The apoAII helical peptides were also inactive, as were the helical amphipathic peptides derived from apoJ, with the exception of apoJ 311-329, which showed slight yet statistically significant antiviral activity (83% inhibition at 40 μM; P < 0.01).

**Table 1:** Antiviral and antibacterial activity of apolipoprotein-derived peptides.

Category	Apolipoprotein	Peptide	Amino Acid Sequence	IC50 Concentration (μM)		
				
				HSV1	*P. aeruginosa*	*S. aureus*
Amphipathic α-helical domain derived	AI	apoA1-H4	PYLDDFQ**KK**WQEEMELY**R**Q**K**VE	-	-	-
		apoA1-H6	PLGEEM**R**D**R**A**R**AHVDAL**R**THLA	-	-	-
		apoA1-H7	PYSDEL**R**QRLAA**R**LEAL**K**ENGG	-	-	-
		apoA1-H8	ARLAEYHA**K**ATEHLSTLSE**K**A**K**	-	-	-
		apoA1-18A	DWL**K**AFYD**K**VAE**K**L**K**EAF	36	-	-
		apoA1-consensus	PVLDEF**R**E**K**LNEELEAL**K**QKM**K**	-	-	-
	
	AII	apoAII 18-35	VTDYG**K**DLME**K**V**K**SPELQ	-	-	-
		apoAII 18-30+	VTDYG**K**DLME**K**V**K**EWLNS	-	-	-
	
	J	apoJ151-170	HMLDVMQDHFS**R**ASSIIDEL	>40	-	-
		apoJ219-236	NFHAMFQPFLEMIHEAQQ	-	-	-
		apoJ311-329	LQVAE**R**LT**RK**YNELL**K**SYQ	-	-	-
		apoJ409-424	**K**FMETVAE**K**ALQEY**RK**	-	-	-

HSPG/heparin binding domain derived	B	apoB1000-1016	**R**ALVDTL**K**FVTQAEGA**K**	-	-	-
		apoB(3359-3367)dp	**R**LT**RKR**GL**KR**LT**RKR**GL**K**	>40	-	-
	
	E	apoE128-149	QSTEEL**R**V**R**LASHL**RK**L**RKR**LL	36.5	54	-
		apoE(141-149)dp	L**RK**L**RKR**LLL**RK**L**RKR**LL	16.5	2.5	9
		apoE141-162	L**RK**L**RKR**LL**R **DADDLQ**KR**LA	>40	-	-
		apoE(150-158)dp	**R**DADDLQ**KRR**DADDLQ**KR**	>40	-	-
		apoE(211-219)dp	GE**R**L**R**A**R**MEGE**R**L**R**A**R**ME	>40	-	-
		apoE(213-221)dp	**R**L**R**A**R**MEEM**R**L**R**A**R**MEEM	>40	-	-
	
	H	apoH(281-288)dp	C**K**N**K**E**KK**CC**K**N**K**E**KK**C	-	-	-

Anti-HSV1 activity was assessed by plaque reduction assay, antibacterial assays were carried out using turbidity assessment of serial dilutions of peptide inoculated with bacteria. In both cases concentrations yielding 50% inhibition of growth obtained from the plots (averages for several experiments are shown here). A dash indicates no detectible activity. Cationic amino acids are shown in bold.

In the second group of peptides, apoEdp - used as a positive control - was active against all three organisms, as found in our previous studies. The short receptor binding sequence when presented within the previously untested non-tandem-repeat peptide apoE(128-149) also showed some antiviral activity, and weaker activity against *Pseudomonas aeruginosa*. Our earlier reports showed that the nonomer peptide apoE(141-149) is inactive, most likely reflecting its lack of structure [9]. Together these data suggest the receptor binding region in the longer apoE(128-149) fragment may adopt a conformation more like that found in full-length apoE, and are consistent with a previous study reporting some antibacterial activity in the much longer apoE fragment apoE(133-162), which declined in shorter fragments [22].

The tandem repeat peptides derived from the second apoE heparin binding domain also showed antiviral activity, however this was far less than that for those derived from apoE(141-149), perhaps reflecting the presence of acidic residues in apoE(211-219) amongst basic ones (we previously reported that introduction of acidic residues into the apoEdp sequence abolished activity [9]).

**Figure 1:**
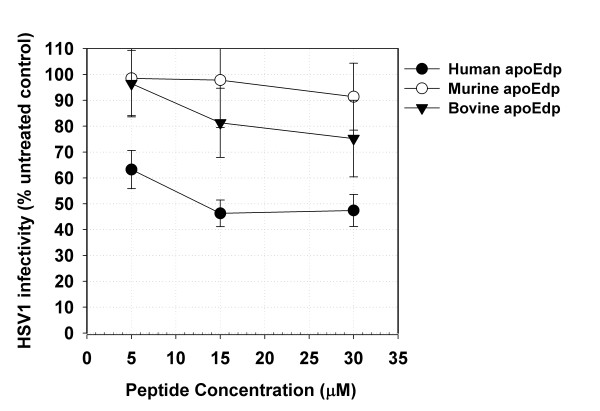
**Anti-infective activity of alternative apoE(141-149) tandem repeat peptides derived from the human, murine and bovine apoE sequences**. HSV1 infectivity after treatment with peptides derived from the human, mouse and bovine apoE141-149 sequence, shown relative to that for untreated controls. Human apoEdp had activity significantly different from untreated wells (P < 0.001) unlike the murine and bovine equivalent peptides. Typical data are shown here; bars indicating standard error.

We also tested peptides equivalent to human apoEdp derived from the murine and bovine apoE sequences. Interestingly both peptides showed significantly less antiviral activity than apoEdp (Figure 1), and no antibacterial activity (data not shown), perhaps reflecting the substitution of four of the Leu residues in the human apoEdp peptide for Met, and disruption of the pattern of charged residues within apoEdp, both of which have previously been found to decrease the activity of apoEdp [9], and suggesting that apoE may not affect viral infection in those mammals which do not share the human apoE141-149 sequence.

Activities in the receptor binding regions derived from apolipoproteins other than apoE were limited (Table 1). The tandem repeat peptide derived from the heparin binding region of apoH, was inactive, perhaps surprising given the high numbers of cationic residues, as was apoB(1000-1006). However the peptide we derived from the apoB LDLR binding region B also showed some antiviral activity (IC75 concentration was 38 μM (95%CI 35.1 - 40.9 μM)).

In conclusion, helical structure, amphipathic character, or a high proportion of basic residues does not on its own appear to be sufficient to result in anti-infective activity in these apolipoprotein-derived sequences. The most active sequence identified from the apolipoproteins not previously linked to anti-infective peptides was apoB(3359-3367)dp (apoBdp), which has some similarity with apoEdp.

### ApoEdp and apoBdp activities

**Figure 2:**
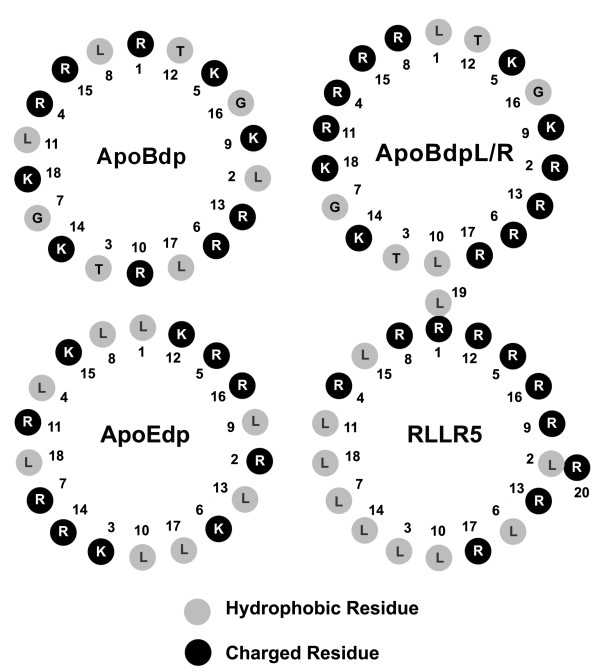
**Edmondson helical-wheel diagrams showing charge distribution of apoE and apoB derived peptides in α-helical conformation**. Charged residues in apoEdp, apoBdp and apoBdpL/R do not show amphipathic distribution (like for example the lytic peptide RLLR5), though are arranged in a bipolar asymmetric pattern which is more pronounced in apoEdp and apoBdpL/R.

**Figure 3:**
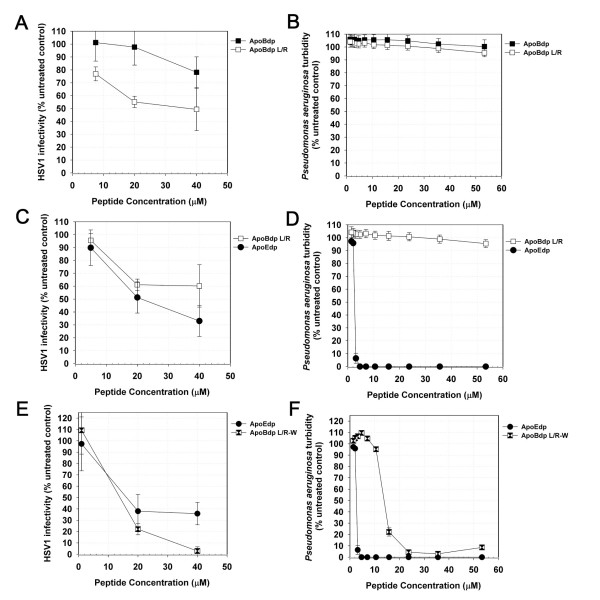
**Antiviral and antibacterial activity of apoBdprelated peptides**. (A) A peptide consisting of a tandem repeat peptide of the naturally occurring apoB3359-3367 sequence has limited antiviral activity which is enhanced in the apoBdpL/R modified sequence, though (B) antibacterial activity was not measurable for both. (C) ApoBdpL/R has comparable antiviral activity to apoEdp though (D) does not share its strong antibacterial activity. (E) The Trp-substituted apoBdpL/R-W peptide has stronger antiviral activity and (F) shows similar antibacterial activity. Values shown are HSV1 infectivity after plaque reduction assay in Vero cells or turbidity of *Pseudomonas aeruginosa; *typical data are shown; bars indicate standard error.

To test whether the activity of the apoB-receptor binding region peptide might be easily enhanced and also possibly related to the activity of the apoE-receptor binding region peptides reported previously, we modified the sequence systematically resulting in mutant peptides which more closely resembled apoEdp. The first such peptide - apoBdpL/R - featured two modifications - reversal of the initial Arg and Leu residues of the apoB3359-3367 nonomer sequence, and substitution of the Leu residue (3366) for Arg. These modifications increased similarity with apoEdp, without modifying the position of the repeated basic Arg-Lys-Arg motifs (found in positions 5,6,7 and 14,15,16 in apoEdp, and in positions 4,5,6 and 13,14,15 in apoBdp). Helical wheel diagrams showed that these changes altered the pattern of charge around the apoBdp α-helix. In apoBdp, charge is evenly distributed around the helix, whereas in apoEdp and apoBdpL/R charge was distributed in a symmetrical yet bipolar fashion. Notably the charge distribution of both peptides remained distinct from the highly asymmetric lytic peptide RLLR5 [23] (Figure 2). We found that the antiviral activity of apoBdpL/R was significantly greater than that of apoBdp (P < 0.002), implicating the bipolar symmetry found in apoBdpL/R (and apoEdp) as a potential mediator of antiviral activity (Figure 3A). ApoBdpL/R did not however have any measurably greater activity against *Pseudomonas aeruginosa *than apoBdp (Figure 3B).

### Aromatic substitutions of apoBdpL/R

**Figure 4:**
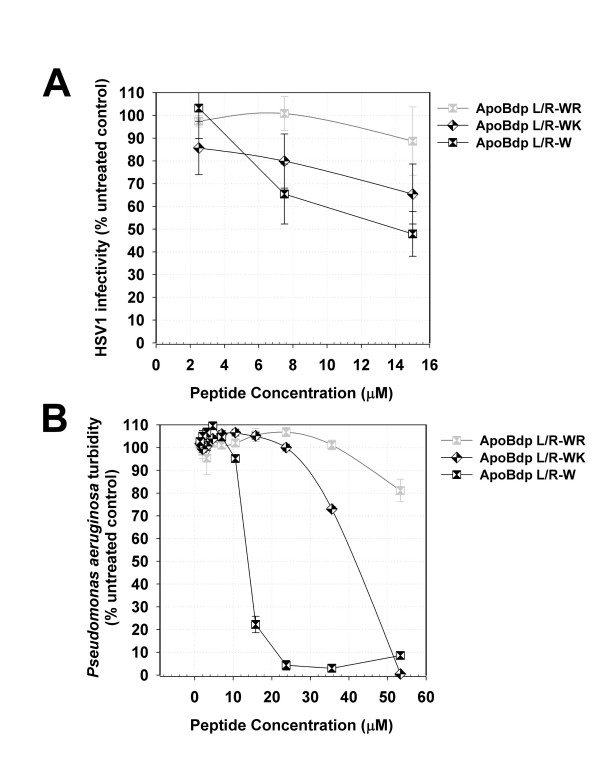
**Reduction of antiviral and antibacterial activity of apoEdpL/R-W peptides after alteration of Arg-Lys-Arg repeated motif**. Substitution of the repeated Arg-Lys-Arg motif within apoBdpL/R-W for Arg-Arg-Arg (apoBdpL/R-WR) or Lys-Lys-Lys (apoBdpL/R-WK) reduces (A) antiviral activity or (B) antibacterial activity. Values shown are HSV1 infectivity after plaque reduction assay in Vero cells or turbidity of *Pseudomonas aeruginosa; *typical data are shown; bars indicate standard error.

We previously reported that substitution of Leu residues within apoEdp with aromatic amino acids resulted in peptides with more potent and broader anti-infective activity [9]. We therefore tested whether similar substitutions within the apoBdpL/R sequence might have similar effects. The peptide apoBdpL/R-W - in which all non-basic residues were substituted for Trp - indeed had more potent antiviral activity (Figure 3C) and clear antibacterial activity (Figure 3D), similar to that we previously reported for apoEdpL-W. One common theme linking these two peptides is the double Arg-Lys-Arg motif separated by six residues. We therefore examined whether substitution of these triplets with apoEdpL/R-W with alternative basic triplets would alter activity. Surprisingly we found that both antiviral activity and antibacterial activity were diminished by the relatively subtle substitution of these triplets with either Arg-Arg-Arg or Lys-Lys-Lys (Table 2 and Figure 4). To test whether activity correlated with alpha-helical structure, we obtained far-UV circular dichroism spectra for several Trp-containing apoBdp derived peptides. However structure could not be determined from the spectra, likely due to interference from the aromatic groups (data not shown).

**Table 2:** Amino Acid Sequences of ApoE and ApoB-derived Peptides.

Peptide	Amino acid sequence																	
ApoEdp	L	**R**	**K**	L	**R**	**K**	**R**	L	L	L	**R**	**K**	L	**R**	**K**	**R**	L	L
Murine ApoEdp	M	**R**	**K**	L	**R**	**K**	**R**	L	M	M	**R**	**K**	L	**R**	**K**	**R**	L	M
Bovine ApoEdp	L	**R**	**K**	L	P	**K**	**R**	L	L	L	**R**	**K**	L	P	**K**	**R**	L	L
ApoBdp	**R**	L	T	**R**	**K**	**R**	G	L	**K**	**R**	L	T	**R**	**K**	**R**	G	L	**K**
ApoBdp L/R	L	**R**	T	**R**	**K**	**R**	G	**R**	**K**	L	**R**	T	**R**	**K**	**R**	G	**R**	**K**
ApoBdpL/R-W	W	**R**	W	**R**	**K**	**R**	W	**R**	**K**	W	**R**	W	**R**	**K**	**R**	W	**R**	**K**
ApoBdpL/R-WK	W	**R**	W	**K**	**K**	**K**	W	**R**	**K**	W	**R**	W	**K**	**K**	**K**	W	**R**	**K**
ApoBdpL/R-WR	W	**R**	W	**R**	**R**	**R**	W	**R**	**K**	W	**R**	W	**R**	**R**	**R**	W	**R**	**K**

Sequences of peptides derived from the apoE(141-149) and apoB(3359-3367) receptor-binding regions; cationic amino acids are shown in bold.

**Figure 5:**
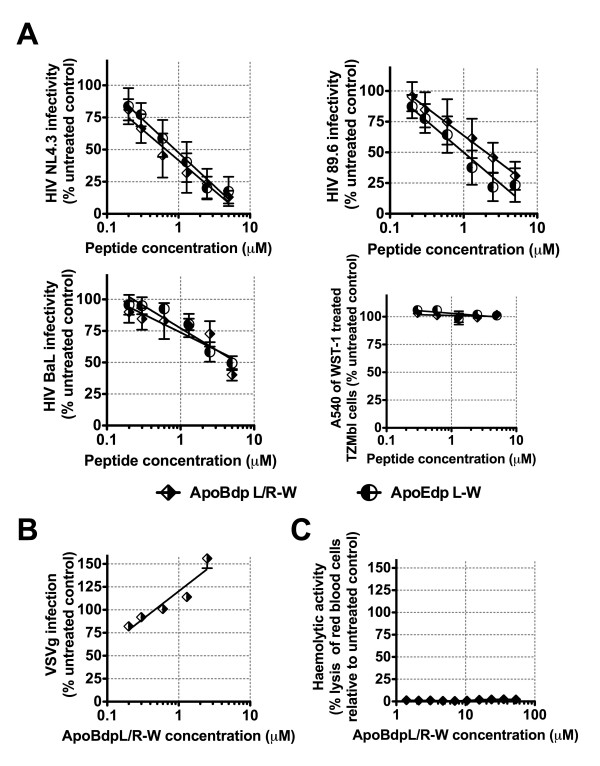
**Anti-HIV activity of the apolipoprotein B derived peptide apoBdpL/R-W**. (A) ApoBdpL/R-W has potent activity against a number of HIV strains, including both CXCR4 and CCR5 co-receptor using strains, similar to that we previously reported for apoEdpL-W. Experiments were performed by treating TZMbl cells with peptide prior to introducing the CXCR4 using HIV strain HIV NL4.3, the CCR5 using HIV strain BaL, and the dual co-receptor using HIV strain 89.6. Values show level of HIV infection (in RLU) relative to untreated control; bars indicate standard errors. No effect was seen on growth of TZMbl cells over the concentration range tested against HIV. ApoBdpL-W does not inhibit VSV-g infection (B) or cause haemolysis (C) suggesting its activity is relatively selective.

Our previous report showed that apoEdpL-W has antiviral activity against both CXCR4 and CCR5 co-receptor using strains of HIV, yet does not show mammalian cell toxicity or haemolytic activity (at concentrations over ten fold the IC50 concentration against HIV). We therefore examined the anti-HIV activity of apoBdpL/R-W to test whether this more potent apoBdp related peptide would show similar properties, supporting its potential as a lead for HIV therapeutics or microbicides. Figure 5 shows that HIV strains NL4.3, 89.6 and BaL were all similarly strongly inhibited by apoBdpL/R-W with IC50 concentrations ranging from 600 nM (NL4.3) to 5 μM (BaL), with these being very similar to those for apoEdpL-W. Concentrations of apoBdpL/R-W at the same levels had no detectable toxicity against TZMbl cells by the WST-1 assay and like apoEdp and apoEdpL-W were non-haemolytic suggesting the anti-HIV activity was relatively selective. This was further supported by the finding that apoBdpL/R-W had no inhibitory activity on a VSV-G pseudotyped virus at these concentrations, also as we previously found for apoEdpL-W [9].

## Discussion

Many of the peptides derived either from apolipoprotein amphipathic α-helical or receptor-binding regions did not show anti-infective activity. Peptides derived from apoA1 had previously been reported to inhibit HSV1 and HIV infection *in vitro *[11,20,21], and although we detected antiviral activity in the consensus domain peptide 18A previously found to be active, we did not obtain evidence of antibacterial activity for this peptide. This is consistent with this peptide's reported selective mechanism of action, involving inhibition of fusion of viral particles with cellular membranes and cell syncytiumformation [11,20,21]. None of the naturally occurring apoAI amphipathic helices had anti-infective activity, suggesting that the antiviral activity previously reported for full length apoAI is not localised to a specific helix. Interestingly one naturally occurring amphipathic helical peptide did show antiviral (but not antibacterial) activity, however this peptide was derived from apoJ rather than apoAI; future studies might clarify whether full-length apoJ itself directly inhibits infection, as is the case for apoAI. All other anti-infective activity we detected was associated with either the HSPG receptor binding regions of apoE (mostly the main receptor binding region apoE(141-149)) or the similar HSPG binding region B of apoB. Unlike the activity of the α-helical amphipathic peptides (from apoAI and apoJ), these receptor binding domain peptides showed broader activity, which appeared to increase for the apoB peptides when charge distribution was altered to resemble the bipolar symmetrical distribution found in the apoE peptides (though with none of these peptides showing amphipathic charge distribution).

It is notable that the presence of cationic residues per se does *not *appear to be sufficient on its own for anti-infective activity in apolipoprotein-derived peptides. Although the apoE and apoB tandem repeat peptides contained the highest number of cationic residues (10-12 per peptide), some of the other peptides had a comparable number - e.g. apoE(141-162) and apoH(281-288) with eight cationic residues each - yet these were inactive or almost inactive. Additionally we found that the precise pattern of cationic residues could strongly affect activity. Modification of the Arg-Lys-Arg repeat in apoBdpL/R-W dramatically reduced both antiviral and antibacterial activity, suggesting these repeated triplet motifs are core to the broad anti-infective activity of both the apoEdp and apoBdp peptide families.

A further similarity between apoEdp and apoBdp related peptides was the effect of substitutions involving aromatic residues on activity; Trp-substitution resulted in peptides with much greater efficacy than unsubstituted peptides, with this being likely to reflect greater membrane perturbation by Trp-substituted peptides, as we previously reported for Trp-substituted apoE peptides. Trp residues have bulky and hydrophobic side chains, likely to interact strongly with non-polar membrane components. The combination of positive charge and hydophobicity found in the apoEdpL-W and apoBdpL/R-W suggests such peptides may disrupt membrane systems, and indeed we previously reported this for apoEdpL-W [9]. Nonetheless not all such peptides show anti-infective activity (apparent from the relative inactivity of some derivatives of apoBdp and apoEdp [9]), suggesting the precise distribution of such residues within the sequence influences the extent of membrane disruption. Additionally the low haemolytic activities and mammalian cell toxicity of apoEdpL-W and apoBdpL/R-W suggests membrane composition also influences the extent of perturbation.

Our finding that (like apoEdpL-W) apoBdp L/R-W had broad activity against herpes viruses and different HIV strains yet minimal cytotoxicity or haemolytic activity, supports its use as a lead for the development of microbicides or HIV therapeutics. An important next step in assessing the potential of such peptides would be to confirm their activity *in vivo*, as has recently been carried out for the related peptide apoEdp [10].

## Conclusions

We have used two approaches to design candidate anti-infective peptides related to human apolipoproteins, based either on sequences from amphipathic α-helical regions or receptor binding regions of apolipoproteins. This strategy uncovered antiviral activity in a peptide derived from apoJ, in a peptide derivative of the second heparin binding region of apoE and in derivates of the cationic HSPG receptor binding region B of apoB. Thus both strategies led to the identification of anti-infective peptides, though such activity was not widespread amongst the sequences tested. Although the strength of activity in those sequences identified appeared limited, in the case of the apoB peptides at least, this was amenable to increase by generating mutant peptides with altered charge distribution. The resulting peptides more closely resembled the previously reported antimicrobial peptide apoEdp, and also like apoEdp, activity increased when aromatic substitutions were made. The Trp-substituted peptide apoBdpL/R-W may have similar utility as a lead compound for HIV prophylactics or therapeutics, akin to that we previously reported for apoEdpL-W. The breadth of activity across HIV strains and herpesviruses, and the possibility of viral membrane being targeted by the agents provides a rationale for apoBdp derived peptides proving less likely to lead to the generation of viral resistance, compared with existing drugs. Our data support the screening of further apolipoprotein-derived sequences to obtain additional anti-infective peptides derived from the human proteome, and the development of mutant peptides related to the natural sequences with enhanced activity.

## Methods

### Cell cultures

Vero cell cultures were maintained in Eagle's minimum essential medium (EMEM) supplemented with 10% (v/v) fetal bovine serum (FBS), 2 mM L-glutamine, penicillin (100 IU/ml) and streptomycin (100 μg/ml), referred to as growth medium. TZM-bl/CD4/CXCR4 or TZM-bl/CD4/CCR5 cells were maintained in growth medium (DMEM) [24]. Growth medium containing only 2% or 0.5% FBS is referred to as '2% medium' or '0.5% medium'.

### Microorganisms

HSV1 stocks (strain SC16 provided by Prof. Roy Jennings, Sheffield University) and herpes simplex virus type 2 (HSV2) stocks (clinical isolate originally provided by Prof. Anthony Hart, Liverpool University) were prepared in Vero and HEp2a cells respectively [8]. HIV-1 stock BaL was prepared in human peripheral blood mononuclear cells stimulated with phytohemagglutinin and interleukin-2. NL4.3 and 89.6 stocks were prepared by transfecting 293T cells with the relevant molecular clones. Virus containing supernatants were harvested 48-72 hours post-transfection. Bacteria were grown by inoculating Luria-Bertani (LB) broth with either *Pseudomonas aeruginosa *(ATCC strain 9027) or *Staphylococcus aureus *(ATCC strain 6538P) (Oxoid) [8].

### Peptides

Peptides were obtained commercially (Alta Bioscience, UK) having been synthesized using 9-fluorenylmethyl carbamate chemistry and purified by high-performance liquid chromatography as described previously [8]. For peptides apoEdp and apoBdpL/R-W, peptide weight was confirmed by amino acid analysis. Peptide stocks were solubilised in phosphate buffered saline (PBS) or growth medium at 400 μM, aliquoted and stored at -80°C.

### Anti-infective and Cytotoxicity Assays

#### Herpes virus plaque reduction assays

Vero or HEp2a cells were grown to confluency in 24-well plates, and were inoculated with 90 plaque forming units (PFU)/well HSV1 or HSV2 in 0.5% medium, containing various concentrations of peptide. After 1 h this was removed, and 0.2% high viscosity carboxymethylcellulose in 1% medium added. After a further 2 d incubation, cells were fixed (formal saline) and stained with carbol fuchsin, before plaques were enumerated. The level of viral infection was expressed as a proportion of untreated control and plotted against peptide concentration, before calculation of peptide concentrations which inhibited infection by 50% (IC50 concentrations).

#### HIV inhibition assays

Dilutions of peptide were added to TZMbl cells which had been plated at 8 × 10^3 ^cells per well the previous day. Peptides were incubated with the cells for 30 min before addition of 20,000 relative light units (RLU) of the test virus (with additional peptide to maintain peptide concentration). Cells were washed 2 h post infection and then incubated for 72 h. Renilla luciferase expression was detected using the Bright-Glo™ Luciferase Assay System (Promega) according to manufacturers' instructions [25].

#### Antibacterial Assays

A microdilution method was used [8]: paired dilutions of compounds in LB broth, arranged in 96-well plates were inoculated with around 1 × 10^5 ^CFU *Pseudomonas aeruginosa *(ATCC 9027) or *Staphylococcus aureus *(ATCC 6538P). After overnight incubation at 37C, absorbance at 620 nm (A620) was assessed, and the IC50 concentrations determined.

Cytotoxicity assessment - Vero cells growing in 96-well plates were treated with various peptide concentrations. After 48 h incubation, 25 μl 3-(4,5-dimethylthiazol-2-yl)-2,5-diphenyltetrazolium bromide (MTT) in 0.5% medium was added (1 mg/ml final concentration), and cells incubated for 2 h. Growth medium was then carefully removed and formazan crystals solubilised in 100 l of DMSO, prior to reading absorbance at 570 nm (A570). TZMbl cytotoxicity was assessed using the WST-1 cell proliferation reagent (Roche). Experiments were performed as for the anti-infectivity assay, WST-1 was diluted 1/10 into each well at various time points and cell proliferation was measured by absorbance at 450 nm.

#### Haemolytic Assays

Freshly washed human red blood cells were added to peptides diluted in PBS in 96-well plates (20 × 10^6 ^red blood cells were added per well). After 2 h incubation at 37°C, plates were centrifuged (3000 g, 5 min) and 80 μl of supernatant transferred to further 96-well plates containing 0.75% ammonium hydroxide in distilled water. After assessment of absorbance at 540 nm (A540), concentration of peptide which resulted in 5% or 50% haemolysis (referred to as EC5 and EC50 concentrations) were calculated (100% haemolysis was considered to be the average A540 for red blood cells treated directly with 0.45% ammonium hydroxide).

### Data Analysis

For anti-infective assays, activity was expressed as % reduction relative to control. Standard error was calculated using a special case of Fieller's Theorem, and significance assessed using ANOVA.

## Authors' contributions

BAK carried out antibacterial experiments. IH carried out anti-HIV experiments and together with ÁMK assisted with study design. CBD carried out anti-HSV1 and mammalian cell experiments, designed test peptides, coordinated the study and drafted the manuscript. All authors read and approved the final manuscript.
